# Learning from missteps: Potential of transcranial electrical stimulation in neuropsychological rehabilitation

**DOI:** 10.1111/jnp.12425

**Published:** 2025-03-23

**Authors:** Carlo Miniussi, Maria Concetta Pellicciari

**Affiliations:** ^1^ Center for Mind/Brain Sciences—CIMeC University of Trento Rovereto Italy; ^2^ Department of Human Sciences LUMSA University Rome Italy

**Keywords:** cognitive plasticity, cognitive training, home‐based applications, neuropsychological rehabilitation, non‐invasive brain stimulation, personalised precision neuromodulation, psychological traits, state‐dependent effects, transcranial direct current stimulation, transcranial electrical stimulation, variability

## Abstract

Transcranial electrical stimulation (tES) holds promise for neuropsychological rehabilitation by leveraging the brain's inherent plasticity to enhance cognitive and motor functions. However, early results have been variable due to oversimplified approaches. This manuscript explores the potential and complexities of tES, particularly focusing on a protocol defined transcranial direct current stimulation as a reference model for all tES protocols, emphasising the need for precision in tailoring stimulation parameters to individual characteristics. By integrating intrinsic (i.e. the neuro‐physiological system state) and extrinsic factors (i.e. experimental set up), highlighting the critical role of state‐dependent effects and the synergy with cognitive tasks, we aim to refine tES protocols. This approach not only addresses the complexity of the brain system (as defined by its current state) but also highlights the importance of collaborative research and data sharing to understand the underlying mechanisms of tES‐induced changes and optimising therapeutic efficacy. Emphasising the integration of tES with targeted tasks and clearer hypotheses, this work underscores the potential for more effective neurorehabilitation strategies.

## INTRODUCTION

In the field of neuropsychological rehabilitation, the quest for innovative interventions to exploit the brain's inherent capacity for change has taken central stage. In recent decades, the prospects of this area have expanded to include an assortment of tools, starting from the implementation of ‘traditional’ cognitive rehabilitation methods into computer‐assisted techniques. These are complemented by interventions such as non‐invasive brain stimulation (NIBS), aerobic exercises, pharmacological and nutraceutical approaches (e.g. Brioschi Guevara et al., 2021; Erickson et al., 2011; Maggio et al., 2023; Zhao et al., 2021). Important to neuropsychological rehabilitation is the consideration of factors like accessibility, feasibility, scalability, resource efficiency and cost‐effectiveness when selecting the appropriate method. Among the emerging neurotechnological approaches in NIBS, low‐intensity transcranial electrical stimulation (tES) and particularly a protocol‐defined transcranial direct current stimulation (tDCS), have gained significant attention as an adjunctive therapy over the past two decades. This interest arises from the extensive experimentation and numerous clinical trials suggesting its potential (Brunoni et al., 2012). tDCS offers a promising method to modulate cortical excitability for studying and enhancing cognitive and motor functions [*Note: Given the complexity of the motor act, we can say that the rehabilitation of central motor functions follows somewhat to the same reasoning as for cognitive functions. Therefore, to some extent, it falls under cognitive functions. Although motor rehabilitation will not be dealt with, what will be said also applies to motor functions*] and promoting neural plasticity (Brunoni et al., 2012; Miniussi et al., 2008; Shin et al., 2015). tES has become a popular technique due to its non‐invasive nature, painless application, high safety profile, low cost, portability, adaptability for add‐on applications and feasibility for conducting double‐blind sham‐controlled studies (Antal et al., 2017). It can be used at home with a controlled protocol (i.e. tele‐medicine, Brunoni et al., 2022), and its stimulation parameters—intensity, duration and electrode montage—are straightforward to set, making it seemingly easy to operate. However, defining the stimulation protocol (i.e. parameters) in relation to specific patient characteristics based on neurological and neuropsychological deficits (but also to inter‐ and intra‐individual variability in health subjects) is where the challenge begins. In fact, embarking on our journey with tES has experienced both strides and stumbles, highlighting that acknowledging our missteps is as crucial as learning from our successes.

Until recently, tES applications have been mainly based on the assumption that placing an electrode over a specific scalp location over the brain area of interest and adjusting the intensity and duration of stimulation in a linear manner would be sufficient. However, over the years, research has shown that this approach is not adequate. The general aim of this manuscript is to highlight the important factors that the scientific community now considers essential when using tES. Rather than providing a systematic review of the clinical literature, this work aims to offer an informed perspective on the critical aspects that should be considered when planning a clinical intervention. Therefore, the manuscript will guide readers through the journey from well‐intentioned but suboptimal procedures to critical considerations of variability, exploring the lessons we are learning and suggesting pathways forward for harnessing the potential of tES to reshape neural‐cognitive circuits. Therefore, the final aim is to provide a generalised theoretical framework, refined through extensive tDCS research, yet flexible enough to be adapted methodologically, independent of specific stimulation parameters (see others tES protocols defined below).

We anticipate that while the inherent variability of tES effects across individuals is a significant factor influencing outcomes, the application of tES in neuropsychological rehabilitation presents unique translational challenges. Rehabilitation settings demand an approach that integrates individualised patient profiles with tailored stimulation protocols. The most pressing challenge in this field is to understand the impact of factors such as pathology‐specific neural dynamics, interactions with ongoing therapeutic interventions and the dynamic state of the brain in response to injury on the tES‐induced effects.

Unlike controlled experimental settings, the variability in patient baselines, including neuroanatomical, neurofunctional, pharmacological and cognitive differences, requires that tES protocols be adapted to optimise the rehabilitation of specific functions. Moreover, as the brain response to stimulation is influenced by homeostatic mechanisms and state‐dependent plasticity, developing targeted, pathology‐driven frameworks for tES application is essential. We well underline in the single sections that future research must therefore emphasise not only the mechanistic understanding of tES but also its practical implementation in clinical rehabilitation, ensuring that stimulation protocols align with the complex and dynamic nature of patient recovery trajectories.

In this manuscript, we highlight the current state of knowledge on tES in respect to the neuropsychology field. This will be achieved by addressing various aspects across distinct sections, each focusing on a specific dimension of the topic. Furthermore, it is important to recognise that most studies investigating the mechanisms and parameters underlying tES effects have been conducted on healthy young individuals. As a result, the present discussion is often based on findings from this population. However, it is crucial to acknowledge that these results, the interpretations and reasoning cannot always be directly extrapolated to non‐young or clinical patient populations, as their neurophysiological and neuropathological profiles may differ significantly. Therefore, an effort must be made to adapt this reasoning to account for the distinct characteristics and needs of clinical populations.

Many reviews already exist on tES/tDCS basic and theoretical mechanisms (e.g. Fertonani & Miniussi, 2017; Stagg & Nitsche, 2011), on safety, ethical and application guidelines (Antal et al., 2017), methodological aspects and training of operators (Woods et al., 2016), neuroenhancement (Antal et al., 2022), recommendations on tDCS efficacy in the clinical context (Fregni et al., 2021; Lefaucheur et al., 2017), inter‐individual (Vergallito et al., 2022) and experimental (Guerra et al., 2020) variability; or issues that should be considered during clinical applications, like study design, blinding, control groups, dropout, surrogate outcomes (Brunoni et al., 2012; Lefaucheur et al., 2017). The reader is referred to these revisions for comprehensive discussion of the different aspects and specific clinical protocols. The reader should also note that there is currently no standard protocol for specific pathologies. Similarly, there is no clear guidance on which parameters to use in clinical practice, as we are still in the early stages of understanding. However, some recent procedures show promise and will be discussed in the section ‘Ideal protocols for tDCS in different pathologies’. It should be underlined, that in this manuscript, we will primarily use the term tDCS, as it is the most commonly used protocol in the literature. However, the principles discussed apply broadly to other protocols as well, with only minor variations specific to each protocol (e.g. transcranial alternating current stimulation—tACS or transcranial random noise stimulation—tRNS). The main assumption is that mechanisms underlying these protocols in the clinical context are essentially the same, that is, polarisation, with the key distinction being the timing of the electrical signal; whether it is applied in a pulsed (e.g. tACS or tRNS) or continuous (e.g. tDCS) manner. The limited use of tACS or tRNS in clinical settings is due to their later introduction compared to tDCS (in 2008) and the need for specialised devices capable of generating alternating currents. The readers should keep in mind that if they choose to use one of these protocols, the underlying principles remain the same as those used for tDCS. The only differences lie in the specific aspects that will be explicitly addressed in the respective sections dedicated to tACS and tRNS.

In the next section, an introduction to the underlying basic aspects of tES is provided, always keeping in mind the action tES has on the brain system: a complex system whose activity relies on neurophysiological and cognitive components.

## tES AS A NEUROMODULATORY TECHNIQUE

### tDCS

The starting point and the most important aspect is the understanding of tDCS underlying mechanisms. The tDCS development within the fields of neurology and cognitive rehabilitation builds upon existing knowledge of animal models and human studies. tDCS has been shown to enhance brain performance and cognitive functions in both healthy individuals and patients (Kuo et al., 2014). Studies on animal models have shown that these improvements are attributed to tDCS's potential to modulate neuronal excitability, neurotransmitter balance and synaptic plasticity, as well as structural changes, demonstrating lasting effects at the neuronal circuit level (Barbati et al., 2022; Cavaleiro et al., 2020).

In principle, tDCS is a specific low‐intensity (subthreshold) tES protocol that produces a constant (i.e. direct) electric field. This electric field increases the likelihood of neuronal membrane depolarization by anodal stimulation or hyperpolarisation by cathodal stimulation, without directly inducing action potentials. Experiments conducted in the last century on the acute effects of DC polarisation on the animal cortex demonstrated that DC polarisation of the cortical surface induces a subthreshold shift in resting membrane potentials, modulating the neurons spontaneous firing rate (Creutzfeldt et al., 1962). These effects outlasted the DC stimulation itself (Bindman et al., 1964). Therefore, neuronal compartments undergo depolarization or hyperpolarisation based on the neurons alignment with the direction of current flow (Bikson et al., 2004). Consequently, tDCS is defined as a neuromodulation technique that influences spontaneous neuronal activity and responsiveness to afferent synaptic inputs (Bindman et al., 1964). Moreover, it has been suggested that anodal tDCS transiently increases intracellular calcium ions, initiating a molecular cascade fundamental to synaptic plasticity mechanisms (Barbati et al., 2022). Therefore, anodal tDCS may affect synaptic modifications within the stimulated area by facilitating the induction of long‐term potentiation (LTP)‐like mechanisms and upregulating neuroplasticity‐related proteins (e.g. Fritsch et al., 2010; Liebetanz et al., 2002; for a review see Cavaleiro et al., 2020). Additionally, tDCS has been shown to induce changes in glial cells and regulate cerebral blood flow, suggesting its impact also on non‐neuronal targets that might favour plasticity (Barbati et al., 2022; Jamil & Nitsche, 2017; Luo et al., 2022; Monai et al., 2016).

Comparable tDCS polarity‐specific effects (i.e. anodal increases and cathodal decreases excitability) have been observed in the human motor cortex (Nitsche & Paulus, 2000, 2001). It has been suggested that primary, acute effects (short‐lasting on‐line effects) are derived from a shift of membrane polarisation (Korai et al., 2021; Nitsche et al., 2003, 2005), changing the balance between excitatory and inhibitory influences. The after‐effects are synaptically driven and are influenced by N‐Methyl‐d‐Aspartate (NMDA) and Gamma‐Aminobutyric Acid (GABA)ergic channel modulation (Nitsche et al., 2003; Stagg et al., 2009, 2011, 2014). Anodal tDCS is hypothesised to decrease GABAergic transmission by inhibiting the synthesis of GABA, while cathodal tDCS has similar effects on glutamate concentrations (Yavari et al., 2018; Zhao et al., 2020), affecting plasticity mechanisms in the stimulated area.

At present, it can be suggested that anodal tDCS primarily enhances and prolongs LTP‐like plasticity, mainly involving the glutamatergic system, with the opposite effect for cathodal stimulation (Melo et al., 2024). In short, tDCS alters the balance between cortical excitation and inhibition, thereby affecting neuronal network physiology. In this regard, it should be underlined that tDCS effects are not limited to stimulated areas but impact other regions that are functionally connected (e.g. Leaver et al., 2022). Moreover, some studies indicate that repeated tDCS applications can lead to cumulative increases in cortical excitability, potentially enhancing LTP‐like plasticity (Ho et al., 2016). To facilitate these clinically relevant LTP‐like effects, it has been suggested that tDCS should be delivered through a multi‐session approach over several days (e.g. minimum 5, usually 10 up to 20 days). This multi‐session strategy is crucial for achieving more substantial and lasting neuromodulatory outcomes (Brunoni et al., 2012; Ke et al., 2023; Lefaucheur et al., 2017; Perceval et al., 2020). Nonetheless, it is important to recognise that specific stimulation parameters and the repetition of a tDCS protocol within a single day can extend after‐effects in a non‐linear way. For example, both standard (1 mA for 15 min) and enhanced (3 mA for 20 min) anodal tDCS protocols in normal subjects have shown significant and comparable increases in cortical excitability. However, when the protocol was repeated within the same day at different intervals, the standard protocol proved to be more effective than the enhanced one (Agboada et al., 2020). This suggests a homeostatic response by the brain system, with inherent boundaries to the obtainable result (Agboada et al., 2020, see also ‘Stimulation parameters’ section below). Likewise, the activity‐dependent mechanisms of synaptic excitability in a targeted brain region can significantly influence the magnitude and direction of its response to a tDCS protocol, sometimes resulting even in a reversed response (e.g. Monte Silva et al., 2013) due to homeostatic metaplasticity (Abraham, 2008). Additionally, given the complexity of brain connections and its state balance, which is adjusted according to the condition we find ourselves in, it is logical to expect that the effects described so far in a didactic manner are actually more complex. Therefore, the specific tDCS‐induced response is not solely determined by the stimulation polarity, intensity and duration. These parameters interact in a complex manner with the induced electrical field on ongoing brain activity, individual neural states and the temporal dynamics of the stimulation.

### tACS

tES includes various protocols beyond tDCS, each named after the mode of electrical current administration. Among the most widely used methods is tACS. This technique delivers alternating currents at specific frequencies; thus, the anode and cathode alternate at each electrode depending on the frequency (Antal et al., 2008). tACS has been suggested to entrain neural oscillations and modulate brain rhythms. It has been used to target cognitive functions linked to brain wave patterns like alpha and beta rhythms (Herrmann et al., 2013). However, questions about the reliability of its entrainment effects remain (Veniero et al., 2017). While the fundamental mechanism of action remains neuronal polarisation, in tACS it occurs at specific intervals, determined by the stimulation frequency. The clinical application of tACS remains limited, partly due to the need for well‐defined experimental hypotheses on oscillatory brain activity and potential oscillopathies. Different frequencies target specific impairments, making precise frequency selection crucial since tACS might restore healthy oscillations. Its effectiveness in stroke rehabilitation (Ding et al., 2025), aging and neuropsychiatric disorders (Grover et al., 2023) has shown moderate potential, but its effectiveness varies depending on the targeted impairment and frequency used (Ding et al., 2025; Grover et al., 2023).

### tRNS

Another tES approach is the tRNS (Terney et al., 2008). This technique applies random electrical coloured noise across a range of frequencies (It is more commonly applied at frequencies ranging from 100 to 1000 Hz, also referred to as high‐frequency (Hf)‐tRNS), inducing facilitatory effects from both electrodes. It has been demonstrated that the induced effects occur without a designated cathode or anode (Pirulli et al., 2016) making this protocol particularly interesting in clinical settings. Moreover, it has been suggested that tRNS is less affected by cortical folding and can stimulate a broader population of neurons (van der Groen et al., 2022). Also, for tRNS, the fundamental mechanism of action remains neuronal polarisation, and it has been shown that it enhances cortical excitability during stimulation. Due to these continuous polarity shifts, it prevents adaptation to a continuous stimulation such as with tDCS (Pirulli et al., 2013). tRNS is a promising protocol that enhances cognitive, sensory and motor functions, with applications in several disorders (ADHD, schizophrenia, stroke rehabilitation, multiple sclerosis, Parkinson's disease, tinnitus and visual disorders) (Herrera‐Murillo et al., 2022), but further controlled trials are needed to integrate tRNS into clinical practice (Herrera‐Murillo et al., 2022; van der Groen et al., 2022).

In the rehabilitation context, both techniques follow the same general principles as tDCS (Fertonani & Miniussi, 2017), primarily relying on neural polarisation to modulate excitability. The key distinction lies in their stimulation pattern: while tDCS provides a constant current, tACS delivers intermittent stimuli, with specific frequency characteristics playing a crucial role in synchronising with neural activity; tRNS delivers intermittent stimuli but at random frequencies inducing a continuous membrane polarity shift. Therefore, the continuous modulation of current polarity in tACS and tRNS (i.e. alternating current) may prevent neural adaptation, as seen with tDCS and specific ion channels (e.g. calcium) may play a critical role in its effects (Terney et al., 2008).

Within this context, it is essential to establish a starting point by recognising that the foundation of neuroplasticity lies in cortical excitability (Paz et al., 2009). This property is intricately linked to the ability of neurons to adjust the strength of their connections, which is fundamental to the brain's capacity. Moving from the neuronal structure to the functional architecture involves assigning a specific role to the connections between neurons (Suárez et al., 2020). Cognitive efficiency hinges on the integration of information with specific direction and timing, highlighting the significance of effective connectivity through neuronal networks. Harnessing the potential of tES to modulate cortical excitability emerges as a strategic approach to fine‐tune cognitive plasticity, such as the brain's ability to adapt its cognitive functions in response to experiences, learning and environmental changes. Cognitive plasticity is crucial for recovering from brain injuries and adapting to new situations. In essence, tES functions as a tool to dynamically shape the neurophysiological landscape, enhancing the brain's adaptability and cognitive flexibility by favouring the activation of the most efficient neural network for solving a given task. This involves optimising effective connectivity within the targeted neural network to enhance performance through improved neuronal communication. The general idea behind using all NIBS approaches in the neurorehabilitation context is that inducing changes in cortical excitability might lead to a recovery or reorganisation of ‘effective networks’ responsible for the impaired function (Yavari et al., 2018). This also means that functional plasticity is the brain's capacity to transfer functions from damaged/depressed to undamaged supporting networks. Therefore, it becomes important to design the adequate stimulation protocol that tackles the proper neural system (via specific brain areas) that will undertake the function (via electrode montage, that will shape the induced electrical fields in specific structures) at the adequate level of stimulation (via current intensity, electrode dimension and duration) (i.e. charge density), number and timing of repetition, to exploit this potential and reach the rehabilitative target. A similar reasoning can be applied if we aim to modulate a maladaptive network by decreasing its activity by cathodal tDCS.

However, one thing we must keep in mind from the outset, in the clinical cognitive neuroscience context, is that we should not see stimulation as a stand‐alone treatment to reach the desired functional outcome. Therefore, tES presents an attractive approach for enhancing the outcomes of various treatments, given its neurophysiological impact on the resting threshold of membranes, which is likely responsible for its synergistic effects. The application of tES on the brain system, when not linked to a specific interventional procedure that fully engages the patient (i.e. the adequate rehabilitation protocol for that patient), may result in mild and short‐lived effects (Blake et al., 2006; Miniussi & Rossini, 2011). In other words, we should view tES as a ‘pedestal’ (i.e. neurally elevating a stimulus can lead to perceptual and neural changes that enhance its neural representation, increasing its salience and significance, see Miniussi et al., 2013) that enhances the likelihood of the rehabilitation protocol emerging effectively because it is facilitated by tES‐induced modulation of cortical excitability and therefore connectivity (i.e. inducing the ‘ideal’ brain state to favour the targeted function). In essence, tES modulates the targeted brain area, but the actual activation of the neural circuit is achieved through the specific cognitive (or other) rehabilitation protocol employed.

Going back to the manuscript introduction, when tDCS started to be used, we thought it would be easy to exploit excitatory or inhibitory stimulation to achieve results. Building upon neurophysiological phenomena documented through direct cortical recordings on experimental animals or indirectly via motor evoked potentials, our approach was grounded on the premise that the polarisation of a cortical area (anodal vs. cathodal) significantly contributes to observed behavioural effects (facilitation vs. inhibition). Additionally, we believed in a linear relationship between the duration of a tDCS session and the subsequent after‐effects on cortical excitability (Nitsche & Paulus, 2001). However, this approach has been critiqued for oversimplifying the transition from physiology to behaviour, without adequately considering the complexity of the underlying neural circuits and brain homeostatic response (Miniussi et al., 2010). As time has unfolded, the ‘stimulation‐dependent’ model we embraced (anodal‐facilitate/cathodal‐inhibit behaviour) has revealed its inherent weaknesses, shifting the field from the peak of overstated expectations in the rehabilitation context (Brunoni et al., 2012; Miniussi et al., 2008) to the level of disillusionment (Horvath et al., 2015; Jacobson et al., 2012). Experimentation with tDCS has showed that even protocols in the motor area that are considered to be well established demonstrate a high degree of variability of results (sometimes only 50% of normal subjects respond as expected see, e.g. Chew et al., 2015; López‐Alonso et al., 2014; Wiethoff et al., 2014).

There are still challenges in accurately characterising the most effective tDCS protocol parameters (Garnett et al., 2015) and the ratio of inhibitory versus excitatory effects over time in different cortical areas (Heimrath et al., 2020). Additionally, several factors have been shown to influence plasticity induction by NIBS as directly evaluated by neurophysiological protocols (e.g. evaluation of motor evoked potential response). For instance, induced plasticity tends to decline with age (Ghasemian‐Shirvan et al., 2020; Perceval et al., 2016) and varies with the time of day, with greater plasticity typically observed in the afternoon compared to the morning (see Ridding & Ziemann, 2010). An important additional factor when evaluating plasticity is sleep. Research suggests that poor sleep or sleep deprivation the night before NIBS intervention significantly impacts the results and is considered detrimental to the efficacy of tDCS protocols (Ebajemito et al., 2016). Hormonal variations, such as those related to sex differences, can also contribute to some of the variability observed in tDCS effects (Kuo et al., 2006). Based on several studies, it has been suggested that cortical excitability in women is higher during the late follicular phase of the menstrual cycle when oestrogen levels peak and lower during the luteal phase due to increased progesterone levels (Krause & Cohen, 2014). Therefore, underlying that there are intrinsic or internal elements (within the individual, i.e. the neuro‐physiological system state) and extrinsic or external elements (experimental setup and history of activity just before the experiment) influencing the final results. In short, we should consider these elements not only in the final results evaluation but also in the implementation of a neurorehabilitative tES protocol.

In this regard, emerging evidence suggests that tES‐induced effects might involve more than just the electric field within the brain, potentially also including the co‐stimulation of cranial nerves in the scalp, which could activate the locus coeruleus–noradrenergic system, possibly leading to neuromodulatory effects on cognitive function (Majdi et al., 2023; van Boekholdt et al., 2021). Moreover, the challenge of establishing precise protocols for assessing the effects in the cognitive domain has also stemmed from the considerable heterogeneity in tDCS parameters and from the initial approach that one‐dose‐fits‐all in moving from healthy (young) individuals to (adult) patients. This variability encompasses electrode placement, stimulation polarity and intensity, just to cite some (Burton et al., 2023). Therefore, drawing from the outcomes of ongoing research studies, meta‐analyses and clinical trials, we accumulated valuable insights into the basic parameters that are at the basis of some weaknesses of tDCS applications (Huang et al., 2017). This knowledge is now guiding investigation towards a more precise targeted parameters method (to delve deeper into the various factors contributing to variability, refer to Guerra et al., 2020).

## PRECISION MEDICINE APPROACHES

Based on this knowledge, the field of NIBS is increasingly moving towards more individualised tES interventions, emphasising personalised approaches for precise neuromodulation (for a review, see Padberg et al., 2021; Soleimani et al., 2023). This involves tailoring stimulation parameters, such as electrode placement, intensity and duration, based on an individual's neuroanatomy, biotype (including genetic factors), baseline cognitive profile and real‐time neurophysiological measures as intervention feedback. By considering all these aspects, along with individual responses to stimulation, we can cluster tES responders to fine‐tune future applications and enhance treatment efficacy. Consequently, we are focused on how individual ‘traits’ and ‘states’ influence tES response. Traits provide a baseline understanding of an individual's typical functioning, while states offer insights into their current condition and responsiveness to stimulation. We are actively exploring these factors to enhance the reliability and effectiveness of tES approaches.

### Genetic variability

Studies have shown that genetic factors contribute to the variability in response to tDCS (Jannati et al., 2017). Some reports have investigated the impact of Brain‐Derived Neurotrophic Factor (BDNF) Val66Met polymorphisms on tDCS responsiveness, suggesting that individuals with the Val/Val genotype might respond better to tDCS compared to those with the Met/Met or Val/Met (Fritsch et al., 2010). On the same line, besides the Catechol‐O‐Methyltransferase—COMT Val158Met polymorphism (COMT) Val/Val and Met/Met, other COMT polymorphisms may also influence tDCS outcomes by affecting dopamine metabolism, which in turn can impact cognitive processes and cortical excitability (Nieratschker et al., 2015). Although this field of research is little explored, it is anticipated that certain genetic factors that carry variations in glutamatergic systems, GABAergic systems, voltage‐gated ion channels or dopamine, acetylcholine, noradrenaline and serotonin transporter will likely influence tDCS outcomes (Nitsche et al., 2006; for a review see Koo et al., 2023). Therefore, incorporating these factors into the design and analysis of studies is essential to minimise variability in outcomes and increase efficacy, assuming such genetic data are accessible and can be shared (Nieratschker et al., 2015).

### Impact of medications

The use of concomitant medications assumed by the patient (e.g. antidepressants, anxiolytics, antiepileptic) can alter neuronal membrane permeability and modulate the conductance of sodium and calcium channels, as well as various neurotransmitter systems, thereby influencing the likelihood of inducing changes in neuronal excitability and plasticity (McLaren et al., 2018; Nitsche et al., 2003). Additionally, the interaction between pharmacotherapy and tDCS is non‐linear; in some cases, what might typically enhance excitability could instead result in inhibition (Kuo et al., 2008). These after‐effects and changes arise from the combination of pathophysiological alterations and drug administration, ultimately modifying the tES‐induced effects (for an overview on tDCS, see Brunoni et al., 2012, Table 1, for transcranial magnetic stimulation—TMS see Ziemann et al., 2015). In general, when stimulating a patient, it is essential to consider not only the pathology but also the potential interaction between medications and the effects of tES. These factors can collectively contribute to increased variability in outcomes, but knowing them, we could control their effects.

**TABLE 1:** Technical parameters reported for ‘treatment’ of chronic aphasia by Fregni et al. (2021).

Aspect	Details
Technical parameters	Intensity: in the range of 1–2 mA
	Duration: in the range of 20–30 min per session
	Daily sessions (in the range of 10–20) over several weeks
Anodal montage	Anodal electrode placed over Broca's area (left IFG) or Wernicke's area (left posterior superior temporal gyrus)
	Cathodal electrode (reference electrode) over the right supraorbital area
Cathodal montage	Cathodal electrode over the right frontotemporal region (right IFG)
	Anodal electrode (reference electrode) over the left supraorbital area
Bilateral montage	Anodal electrode over Broca's area
	Cathodal electrode over its right hemisphere homologue

Abbreviation: IFG, inferior frontal gyrus.

### Neuroanatomical differences

The structure and organisation of an individual brain significantly impact the effects of tDCS (Liu et al., 2018). The tDCS‐induced electric field has a specific spatial interaction with cortical neurons that features another contributor to variability. Brain anatomy, including the size and location of specific brain damage (Datta et al., 2010; Evans et al., 2022; Minjoli et al., 2017; Wagner et al., 2007) or physiological alterations bound to aging (Indahlastari et al., 2020), influences how electrical currents travel through it. Traditionally, tDCS is considered non‐focal, with electrode positioning based on the International 10–20 electroencephalography System. However, modelling the induced electric field in individual subjects has proven crucial for understanding if we are reaching the stimulation targets (Lee et al., 2021). This approach allows for consistent targeting across individuals based on stimulation parameters but also on specific lesion characteristics. Modelling, which defines electrode positioning and configuration (i.e. montage), should be performed for both multi‐ and bi‐cephalic as well as mono‐cephalic montages. Precise electrode location, dimension and arrangement on the scalp influence the specificity and direction of the electrical current (Bikson et al., 2016) and the overall effects (Foerster et al., 2019). Research has shown that even minor shifts in the orientation of a rectangular electrode, in respect to the precentral cortical gyrus, can lead to a reduction of anodal tDCS expected increase in motor evoked potential outcome (Foerster et al., 2019). To address this issue, free modelling software is being developed to account for individual anatomical variability and optimise electrode placement (e.g. https://simnibs.github.io/simnibs/build/html/index.html; Saturnino et al., 2019). Consequently, individualising tDCS electrode configurations is possible (for a review see Hunold et al., 2023). In neuropsychology, accurately identifying and targeting specific brain regions associated with cognitive functions (or regions that vicariate the function) needing rehabilitation is crucial. Selecting optimal electrode placements based on neuroanatomical constraints and individual variability enhances the precision and efficacy of stimulation (Evans et al., 2022).

### Stimulation parameters

Physical parameters are crucial for determining baseline effects in experimental preparations or models. Thanks to the foundational studies (e.g. Nitsche et al., 2005; Nitsche & Paulus, 2000, 2001), we understand the key parameters required to modulate the state of neurons. However, when shifting to stimulating a complex system like the brain, we must consider that the initial state of a subject is not a universal baseline, as seen in experimental preparations, but is specific to each individual. Additionally, the brain is an organ that actively responds to stimulation, engaging mechanisms that aim to maintain homeostasis. In this regard, a further aspect to consider is related to the relationship between the type and dose of stimulation and the strength of the effect or the amounts of electric current to be delivered (dose–response relationships). This implies that the stimulation parameters are key factors. Intensity, duration and timing of tDCS can affect its outcomes but, as anticipated, not necessarily in a predictable way by linear reasoning (Esmaeilpour et al., 2018). As an example: increasing current duration and/or intensity of stimulation does not necessarily induce an increase in the strength of the final effects (Ehrhardt et al., 2021; Ho et al., 2016). It has been demonstrated that increasing stimulation intensity or duration can revert the expected effects, with cathodal stimulation facilitating and anodal stimulation inhibiting the final outcome (Hassanzahraee et al., 2020; Monte Silva et al., 2013; Mosayebi Samani et al., 2019, 2020). For instance, 1 mA cathodal tDCS applied to the motor cortex decreases excitability, while 2 mA application increases excitability, as indexed by changes in motor evoked potential amplitude (Batsikadze et al., 2013). Therefore, effects observed in the primary motor cortex are expected to yield an even more complex picture when we apply stimulation to associative cortices, especially in terms of their behavioural outcomes and interactions within broader neural networks (Narmashiri & Akbari, 2023; Stephens & Berryhill, 2016; Weller et al., 2020). The intensity (1 vs. 2 mA) of anodal tDCS over the dorsolateral prefrontal cortex was found to interact with cognitive load (verbal 1‐, 2‐, 3‐back paradigm), suggesting that cognitive load rather than tDCS intensity could be a decisive factor for effects on working memory (Papazova et al., 2020). In the same way, anodal tDCS over the prefrontal cortex improves performance, but with non‐linear effects on task outcomes: low intensities (.7 and 1 mA) provided the greatest benefit for trained tasks, decision‐making performance, while higher intensities (1 and 2 mA) led to increased performance only in untrained tasks: same task different stimuli (Ehrhardt et al., 2022).

### Inter‐individual variability

We should consider that the dynamics of these effects, often assessed in young subjects, may differ in brains with altered physiology due to aging or specific pathologies (as reported in the ‘Neuroanatomical differences’ paragraph). For example, the decrease in grey matter volume with aging reduces the magnitude of current density in older brains compared to younger ones (Indahlastari et al., 2020; Mahdavi & Towhidkhah, 2018) and the same will be with individual brain lesions. A thorough and precise patient classification process is crucial, requiring a comprehensive assessment of various anatomo‐functional parameters. This evaluation includes considerations such as the specific phase of the pathology (e.g. stroke, distinguishing between acute, post‐acute and chronic stages) and a detailed examination of the degree of clinical impairment (for a review see Grefkes & Fink, 2014). Moreover, in rehabilitation, various theoretical models explore the neurofunctional dynamics of brain disorders, emphasising the importance of individual differences and providing frameworks for tailoring tDCS applications. These models are instrumental in accounting for inter‐individual variability in response to stimulation, as long as we consider the patient state. For instance, the interhemispheric competition model, originally developed for the motor system (Marshall et al., 2000), has also been applied to other functions, such as aphasia (see de Aguiar et al., 2015). This model proposes distinct roles for the ipsilesional and contralesional hemispheres during stroke recovery, with contralesional activity being beneficial in the acute and subacute phases but potentially hindering recovery in the chronic phase (Di Pino et al., 2014). Understanding these evolving concepts across different pathologies and models is crucial for optimising treatment protocols and effectively targeting the underlying neural mechanisms. In a broader vision of tailored tDCS application, if we are able to capture intra‐individual variability, we will automatically address the issue of inter‐individual variability as well.

### Differential effects of baseline performance on tES gains

Given that tES plays a role in modulating synergistic effects to influence the outcomes of other treatments, it is reasonable to anticipate that the subject state, and more specifically the state of the stimulated brain region/network, will also be a pertinent factor to consider. Even basic somatic reflexes (e.g. Golgi tendon reflex) can vary in intensity or direction of response when the triggering stimulus remains constant but the postural condition changes, changing the neurophysiological system configuration and therefore equilibrium (e.g. the wiping reflex Fukson et al., 1980); it stands to reason that more complex functions would exhibit similar variability or a better definition would be adaptability. Therefore, a further element that has emerged over the years is that individuals may respond differently to various stimulation parameters, based on the ‘system state’ (consider that the state can be intrinsic or extrinsic), that is, one relevant font of sample heterogeneity (Vergallito et al., 2022). The initial cognitive state of an individual may affect their response to tES. For example, individuals with weaker/stronger perceptive or cognitive baseline performance may show varying responses to tDCS intensities (e.g. Benwell et al., 2015) or experience differential performance gains (Krebs et al., 2021; Sarkar et al., 2014). This phenomenon suggests that baseline system abilities can significantly influence the efficacy of neurostimulation interventions, highlighting the importance of personalised treatment protocols.

### Psychological traits

Similar to the state‐dependent effects, the psychological state during tES can significantly impact outcomes (e.g. Esposito et al., 2021). Factors such as attitudes, expectations, attention, arousal and anxiety traits can influence perception, behaviour and response to tDCS (Schutter et al., 2023). For instance, subjects with higher alertness during tasks did not show performance improvement with anodal tDCS compared to those with normal alertness levels (Esposito et al., 2022). Similarly, anxiety traits negatively affected facilitation (Esposito et al., 2021), underscoring the role of psychological factors in shaping tES outcomes (for a review, see Schutter et al., 2023).

On the other hand, a clinical condition arising from a dysfunctional activity state of the brain system can drive stimulation with an immediate impact on symptom severity (Scangos et al., 2021 for closed‐loop therapy with Deep Brain Stimulation).

The brain is a dynamic electrochemical system that continuously adapts its response to a changing environment. When applying a weak neuromodulatory current, like tES, we should tune the stimulation parameters considering the global system state. In short, an all‐inclusive view of the individual encapsulates various intrinsic and extrinsic factors, like current neurophysiological, psychological and environmental conditions at the time of stimulation. A precise evaluation of the patient's general state and especially the degree of deficit prior to treatment is becoming a fundamental aspect to determine how the person will respond to neuromodulatory interventions.

### Complementing tES with therapeutic intervention

Any development in this field must consider the integration of tES with other therapeutic interventions. Combining tES with cognitive training, training exercises or rehabilitation programs may produce synergistic effects and enhance the overall efficacy of rehabilitation programs (Burton et al., 2023; Diana et al., 2024). Therefore, the timing of stimulation of an area and task execution, whether sequential or concurrent stimulation, is also an issue to be considered with attention (Fertonani et al., 2010; Kuo et al., 2008). This underexplored aspect becomes important based on when we want to intervene relative to the cognitive process, that is, off‐line priming versus on‐line boosting versus off‐line consolidation or interference. Priming involves pre‐exposure to tES potentially enhancing subsequent performance; boosting refers to enhancing performance during task execution through tES, while consolidation or interference affects the retention of learned information post‐task, with off‐line stimulation either consolidating or disrupting synaptic memory. Additionally, another aspect that we should consider is the timing of sequential or concurrent stimulation of different areas involved in the task (Kaminski et al., 2024). Investigating the benefits of coordinating the stimulation of interconnected brain areas involved in specific cognitive functions could theoretically enhance network‐level effects.

Engaging in cognitive tasks during or immediately after tES may capitalise on increased cortical excitability and plasticity, promoting more robust learning and neural adaptations. However, we must keep in mind what has been said up to this point and evaluate what the effects of the cognitive intervention alone are (e.g. Bahar‐Fuchs et al., 2013). Since tES effects on overall cortical excitability might impact the excitation/inhibition network balance and so does the task (if it implies learning) the final outcome will be a combination of these effects. It has been shown that applying anodal stimulation, which increases the excitability of the stimulated area during a task like motor practice that implies learning, leads not to enhanced learning but to interference. However, when the same task was performed at a speed that did not involve learning, the addition of anodal tDCS promoted learning (Bortoletto et al., 2015). A similar result was obtained by administering tDCS before engaging in robotic training that resulted in improvement, whereas applying tDCS during or after training resulted in worsened outcomes (Giacobbe et al., 2013). To achieve improvement through combined treatments, it is essential to maintain a balanced excitatory state within the brain system. Most importantly, the resulting activity must align with the system's functional homeostatic range, ensuring that any induced changes fall within the natural limits of the system equilibrium. This physiological balance is essential for the nervous system to respond effectively to environmental stimuli. While the permanent disruption of the excitation and inhibition ratio can give rise to pathological conditions (Krause et al., 2013; Yizhar et al., 2011).

### The role of task‐induced brain states in predicting neuromodulation outcomes

Inducing a specific brain state through a task or instruction before neuromodulation can provide insights into predicting the final outcome of the intervention. This process leverages the concept of state‐dependent neuromodulation, where the brain's current functional state influences how it responds to stimulation (Silvanto et al., 2007). By engaging patients in specific tasks at an appropriate neural level based on patient condition or providing instructions that induce particular brain states, researchers can create a controlled environment that enhances the predictability of neuromodulation outcomes. For example, tasks that activate targeted neural circuits can help align the brain state with the intended effects of the stimulation, improving the accuracy of outcomes. Considering that cognitive training employs either restorative techniques aimed at improving impaired cognitive abilities by a given network or rehearsal‐based methods, which focus on repeated practice to strengthen specific cognitive domains and networks, we can hypothesise that it is possible to target specific neural circuits underlying these interventions. By identifying and stimulating these circuits, cognitive training can be more precisely tailored to enhance the effectiveness of interventions, thereby promoting targeted neuroplasticity and functional recovery (Martin et al., 2013).

## ADDITIONAL ISSUES

One of the most recent advancements is transcranial temporal interference stimulation (TI or tTIS) (Grossman et al., 2017). tTIS involves the use of two high‐frequency tACS in the kilohertz range, which intersect in a specific region within the brain, creating a low‐frequency modulation at the target site. tTIS has been suggested to reach deeper brain structures with a high focus, making it promising for stimulating subcortical regions and offering new treatment avenues for various clinical conditions (Wessel et al., 2023). Despite several technical details and increased specificity, these tTIS protocols generally might share similar mechanistic effects and critical considerations with the other tES protocols. However, these tES‐specific protocols may target more precise effects/results, and as we will better understand the underlying mechanisms, their use will become even more targeted and effective.

### Transcutaneous vagus nerve stimulation

A small paragraph is needed for an additional emerging technique, that is, transcutaneous vagus nerve stimulation (tVNS). Unlike transcranial methods, tVNS involves stimulating through the skin of the auricular branch (taVNS) or the cervical bundle (tcVNS) at neck level of the vagus nerve using electrical impulses (Farmer et al., 2021). Therefore, this approach does not aim to directly stimulate or modulate brain excitability; instead, its goal is to engage a broader neuromodulatory circuitry via the vagus nerve of the parasympathetic nervous system. The vagus nerve plays a crucial role in modulating brain function and autonomic control to maintain psychophysiological balance, and tVNS has shown promise in enhancing executive functions and attention (Ridgewell et al., 2021). By leveraging this technique, researchers aim to improve outcomes in patients with impairments resulting from several neurological conditions (e.g. epilepsy, migraine, chronic pain, depression, anxiety but see Farmer et al., 2021). Animal studies have suggested that tVNS can enhance activity in the locus coeruleus‐noradrenaline network. However, this mechanism lacks clear evidence in human subjects (Burger et al., 2020). Applying tVNS simplifies electrode placement by targeting the vagus nerve at the ear, minimising concerns about electrode location variability. Nevertheless, the definition of protocol stimulation parameters remains unclear. Additionally, considerations focus on the role of vagus nerve afferents in cognitive modulation, offering a new avenue for clinical outcomes through this approach (Wang et al., 2022).

### Blinding and placebo

Placebo effects and individual beliefs about the effectiveness of the tDCS intervention may contribute to variability in outcomes (Fonteneau et al., 2019; Petersen & Puthusserypady, 2019; Turi et al., 2018). The mere act of participating in a study, and thus being the focus of attention, can influence not only the participants behaviour but also the baseline values of many physiological parameters (Cizza et al., 2014). Placebo effects should be considered as part of the treatment and therefore not discarded a priori if they have a positive influence on the outcome. Nonetheless, in a context where we want to assess the real efficacy of a treatment, we have to ‘measure’ placebo with a sham treatment producing the same then real tDCS cutaneous sensation (adverse effects, for example, itching, tingling, burning—see Fertonani et al., 2015; Turi et al., 2019). tDCS, blinding can generally be obtained for intensities up to about 1.5 mA but note that electrode area, that is, current densities, ramp‐up timing, electrode contact resistance might be relevant factors to modulate blinding perception (see Fertonani et al., 2015). Moreover, when electrode contact (and impedance) changes, such as when an electrode becomes displaced by subject movements, the current‐controlled device may transiently increase the current, causing unpleasant sensations and unblinding the stimulation protocol. This issue can be mitigated by ensuring the robust, motion‐free application of electrodes (Fertonani et al., 2015). Note that these movements are generally minimal if the electrodes are properly secured. Complete displacement is rare; however, in such cases, some devices may cease delivering stimulation due to poor contact caused by increased impedance levels.

The procedure for sham tES involves applying a current for <10 s, with a 10–20 s ramp up and 10–20 s ramp down (higher intensities could benefit from longer rise time), at the beginning and at the end of the treatment to mimic perceptual effects (Ambrus et al., 2012). Pre‐programmed stimulators also allow a double‐blind. When intensities increase over 2 mA (but sometimes even at 1 mA see Greinacher et al., 2019) blinding may be less effective, and in this case, the use of local anaesthetics can solve the issue (EMLA—McFadden et al., 2011). In general, applying topical anaesthetics to the scalp in experimental studies rules out the transcutaneous effects, ensuring that the observed outcomes are attributed to the transcranial neuromodulation. Nonetheless, in all protocols, the perception of these sensations, but also the ability to discriminate between real and sham stimulation, should always be assessed with a dedicated questionnaire and later compared with treatment data. The questionnaire of sensations related to tES, approved and shared by the NIBS community, is reported in table 11 of Antal et al. (2017). Experiencing the perceptual adverse sensations induced by tES on a subjective level may play a role in triggering placebo or nocebo effects.

### Accessibility and feasibility

Treatments that are easier to administer or can be delivered remotely (e.g. tele‐rehabilitation) are often more cost‐effective and widely accessible. Given the importance of the so far underlined individualised protocol, the home‐based and remote applications should follow a more precise approach, developing specific protocols and verifying the adherence by the patients to the adequate procedures. The development of user‐friendly and more effective targeted stimulation devices could lead to increased home‐based and remote applications. Aspects that are currently considered most problematic are remote supervision and accurate electrode placement (Brunoni et al., 2022). Therefore, tES devices with enhanced precision, control and monitoring capabilities, such as artificial intelligence for proper electrode montage, providing feedback to clinicians (Wang et al., 2023) and ideally to patients using home‐based approaches, are crucial for reducing variability in individual responses and increasing efficacy. This could provide greater accessibility to individuals who may benefit from rehabilitation but face challenges in daily accessing traditional clinical settings, but also have a great impact on rehabilitation costs in terms of direct (e.g. clinician time) and indirect costs (e.g. reductions in hospital readmissions, travelling). Digital trials using mobile tES are certainly an approach that will be developed in the coming years, but they are still at an early stage where there is no full consensus among experts on certain issues (Brunoni et al., 2022). The reader interested in digital trials, methodological aspects and available devices can refer to the consensus statement by Brunoni et al. (2022). Some studies, not in the tES field, utilising wearable devices demonstrate that auditory stimulation during sleep with real‐time closed‐loop systems can significantly enhance sleep quality and cognitive performance. A closed‐loop system refers to a physiological approach where real‐time feedback is used to continuously adjust and optimise the system's output, ensuring more precise and adaptive control based on ongoing performance data. The prospect of using tES based on a similar closed‐loop approach, where real‐time feedback from the brain activity is used to adjust the stimulation parameters dynamically, appears promising, as it could offer personalised, adaptive and effective neurointerventions tailored to individual needs and real‐time responses (Lustenberger et al., 2022; Navarrete et al., 2024; Nguyen et al., 2023).

### Training and expertise of practitioners

Proper training is essential for ensuring that tES applications are performed accurately and consistently, as even minor deviations in technique can lead to significant variations in outcomes (Woods et al., 2016). This need for rigorous training is a common challenge across the use of various tools, and tDCS is no exception. Ensuring that researchers, clinicians and technicians are well trained in all aspects of tES application is essential for maintaining consistency in stimulation parameters, electrode placement, safety and overall methodology (Antal et al., 2017; Woods et al., 2016). This comprehensive training helps minimise variability in outcomes and enhances the reliability and validity of findings. By prioritising rigorous training, the field can move towards more standardised and reproducible protocols, ultimately advancing the efficacy and understanding of tDCS in rehabilitation.

### Safety

Even if tES has been considered a safe approach, the exclusion criteria commonly employed for TMS are similarly applicable when considering tES; application should follow the specification reported in table 9 Screening questionnaire for tES in the safety, ethical, legal regulatory and application guidelines by Antal et al. (2017).

### Sample requirements

Over the years, one of the main problems in the robustness of clinical trials has been the number of patients tested, which is always very low, often making the results unreliable due to the great variability. To obtain a level of clinical evidence (Class I) for tES studies, more than 25 participants per group are recommended (Lefaucheur et al., 2017). Surely future tES clinical trials must take this into account and include the appropriate computations necessary to make sample size estimations, which should be done considering the primary study outcome. Clearly, this is not a problem specific to the field of neuromodulation but concerns almost all areas of research (Helwegen et al., 2023).

### Data collection and analysis

To advance this field, it is critical to report all technical details used in the protocol, including a comprehensive list of parameters and settings. Alongside the reporting of stimulation parameters, control conditions and adverse events, documenting confounding variables is equally important. It is also crucial to include and analyse subjects who did not exhibit the expected response, as their data can provide valuable insights into the variability and effectiveness of the treatment. These variables can significantly influence outcomes and must be meticulously accounted for. For a concise list of relevant variables with known impacts on tES outcomes, refer to table 10A in Antal et al. (2017). For a comprehensive list, see table 10B (Antal et al., 2017). Detailed reporting of these elements is crucial for transparent and effective research, allowing for accurate replication and validation of findings across studies.

### Importance of data sharing in neurostimulation research

Sharing data and protocol in a findability, accessibility, interoperability and reusability (FAIR) mode with the research community, even with small sample sizes, is crucial. Moreover, initiatives like the Big NIBS Data Collaboration (https://bignibsdata.com/) highlight the value of such practices. By sharing data and performing collaborative projects, we can obtain critical information that accelerates knowledge advancement, ultimately benefiting patients through improved treatment protocols and outcomes. These collaborative approaches enhance transparency, reproducibility and the collective understanding of neurostimulation effects.

## IDEAL PROTOCOLS FOR tDCS IN DIFFERENT PATHOLOGIES

In an ideal scenario, after outlining all considerations for optimising tDCS use, the manuscript would present the best protocols for different pathologies. Unfortunately, such protocols do not yet exist. The reasons why there are no definitive protocols have been underlined over the manuscript so far. The variability in individual responses, the complexity of brain states and the nuances of each pathology necessitate continuous research to understand the neuro‐physio‐pathological mechanisms and optimise their protocols. It is crucial that this research follows community‐shared logic with clear hypotheses regarding specific pathologies, targeted functions or subcomponents and how results are evaluated.

As an example of such heterogeneity, current findings in Alzheimer's disease do not consistently support the therapeutic efficacy of tDCS (Pellicciari & Miniussi, 2018). This inconsistency arises from methodological issues, such as differences in stimulation parameters, target sites and study design. Data show that improvement can be obtained with either anodal or cathodal stimulation without clear differentiation of site or protocol, raising questions about whether the observed results are directly attributable to tDCS.

However, some studies have focused on providing recommendations for tDCS efficacy according to clinical indications, attempting to identify the protocols that have yielded the most consistent results to date (Fregni et al., 2021; Lefaucheur et al., 2017). These studies aim to standardise tDCS application by extracting evidence‐based protocols from investigational therapies, offering guidance for future clinical applications. Among the various applications for cognitive deficits, the only one providing some evidence has been the treatment of stroke‐related aphasia. Starting from the fact that aphasia is the most studied function, but without differentiation in measures, Fregni et al. (2021) identify a ‘level C’ of possible tDCS efficacy. The reviewed applications aimed to balance interhemispheric competition by enhancing the damaged left hemisphere residual language areas or inhibiting the right hemisphere inhibitory effect (if any!). The studies, mainly focused on chronic aphasia, found that anodal tDCS often improves naming, repetition and conversational content units, though results vary with aphasia type and lesion location. While at present, there is no evidence of improvements in functional communication (Elsner et al., 2019). These parameters are outlined in Table 1, but note that they may not necessarily be the optimal choice; however, they are currently the only ones demonstrating possible efficacy. Additional research has indicated that the long‐lasting effects of these applications can be categorised as having low to medium efficacy (Bucur & Papagno, 2019). Ideally, for the future, all these parameters should be evaluated in the light of the new available information on the factors influencing tDCS efficacy.

It should be emphasised that only a few studies (four) were selected in the final evaluation, although insights were also drawn from other works (Fregni et al., 2021). Given the evidence and levels of recommendation it is suggested that, at present, tDCS in aphasia should only be used as off‐label treatment.

The ideal solution to identify a good starting protocol would involve creating a panel of experts from various fields, not just NIBS, to develop a protocol that can be shared and tested across multiple laboratories. This would allow for the evaluation of its effectiveness and adaptation, based on results. While an ideal protocol has yet to be established, one can construct a suitable protocol by following an algorithm that considers the crucial aspects involved. An illustration of such an algorithm is provided in Table 2.

**TABLE 2:** Possible approach for setting up a transcranial electrical stimulation (tES) protocol.

General algorithm for setting up a tDCS (tES) protocol
Define objectives
Identify the specific cognitive function (subcomponent) or impairment to be addressed considering the specific theory, task to be used and outcome measure(s)
Comprehensive patient assessment
Evaluate individual characteristics such as neuroanatomy (i.e. lesion or altered area), genetic profile, baseline cognitive function, psychological state and relevant biomarkers (as much as possible in terms of cost and benefits)
Select appropriate tDCS (tES) type
Choose between anodal, cathodal or bilateral tDCS (or other tES protocol) based on the targeted cognitive function and underlying theory and neuropathophysiology (state of the to be stimulated area)
Electrode placement/montage
Use neuroimaging and anatomical landmarks to determine precise electrode positioning by modelling, ensuring alignment with the targeted brain region(s)
Determine stimulation parameters
Intensity: Adjust intensity based on modelling and pilot initial responses, aiming for the optimal balance between efficacy and modelling
Duration: Consider varying session lengths and their cumulative effects, adapting as needed based on patient feedback and observed outcomes
Frequency: Determine the optimal number of sessions per week and total duration of the treatment course, using a flexible approach to accommodate individual progress
Each parameter should be adjusted based on individual responses and specific therapeutic goals
Induce specific brain states
Engage patients in specific tasks or provide instructions that induce relevant brain states before or during stimulation
Monitor real‐time physiological and neural responses to adjust parameters dynamically
Integrate with therapies
Combine tES with cognitive training, or rehabilitation programs to enhance synergistic effects
Data collection and analysis
Record patient responses, side effects and cognitive outcomes using standardised questionnaires
Use artificial intelligence and machine learning to analyse data, identify patterns and refine protocols
Iterate and personalise
Continuously adjust protocols based on individual responses and cumulative data
Ensure blinding and placebo control
Implement sham control and blinding techniques to differentiate between real and placebo effects
Training and expertise
Ensure practitioners are well‐trained in tES application, electrode placement and protocols to maintain consistency and reliability
Address safety and ethical considerations
Follow established safety guidelines and ethical standards for tES application
Data sharing and collaboration
Share findings, data and protocols with the research community to enhance transparency, reproducibility, for improvement, validation and collective understanding of tES effects

## MOVING FORWARD WITH ADAPTIVE tES PROTOCOLS

It is becoming increasingly clear that advancements in knowledge necessitate a very precise definition of the patient state before using tES. Researchers are increasingly recognising the importance of tailoring tES interventions to individual characteristics to enhance treatment efficacy. Consequently, titrating stimulation parameters for a certain population or individual is becoming pertinent. Although precise measurements for titrating various parameters are not currently available, we must rely on evaluating and weighing different parameters through specific neuropsychological testing and surrogate biomarkers. This also involves using biomarkers derived from the hemodynamic response with functional magnetic resonance imaging (e.g. Ghobadi‐Azbari et al., 2021), electrophysiological activity measured by electroencephalography (e.g. Chan et al., 2023) to identify optimal neural connectivity or other neuropsychological assessments to pinpoint optimal behavioural performance. Additionally, combining these methods with TMS to evaluate reactivity through motor evoked potentials (e.g. Mosayebi‐Samani et al., 2021) or TMS‐evoked potentials (TMS‐EEG) can help achieve an ideal stimulation protocol, stimulation targets and response to stimulation for a specific individual (Siebner et al., 2022). Advances in neuroimaging and electrophysiological decoding can contribute to personalised open loop treatment plans. For the future, probably the most advanced procedures are related to closed‐loop approaches, combining tES with real‐time neuro feedback, where stimulation devices receive information about the brain activity, to create a closed‐loop stimulation system. This approach allows for dynamic adjustments to the stimulation based on the ongoing neural responses, potentially optimising the intervention instead of simply relying on heuristic rules (Zrenner & Ziemann, 2024). Implementing adaptive algorithms that continuously monitor and adjust stimulation parameters based on individual responses may enhance the effectiveness of tES. This approach allows for real‐time optimisation during the rehabilitation process (note that this technique goes well beyond the standard neurofeedback approach that has been used since last century).

The recent advancements in artificial intelligence for optimising individualised protocols have garnered significant interest due to their promising results (Rajpurkar et al., 2022). The process of detecting relevant patterns and enhancing response prediction through deep neural networks involves several key steps: collecting and pre‐processing biomarker and clinical data, designing and training a deep neural network model, extracting features and recognising patterns from the data and using the trained model to predict treatment responses. These techniques can handle complex, high‐dimensional data, thereby potentially improving the accuracy and efficacy of treatment predictions. Machine learning approaches can be employed to cluster large datasets and classify parameters and responses (e.g. Li et al., 2022), aiding in the development of advanced personalised precision neuromodulation protocols (e.g. Tang et al., 2022). This approach helps identify patterns and predict optimal stimulation parameters for new patients, aiming to achieve targeted stimulation with enhanced electric field intensity and focality (Wang et al., 2023). Additionally, optimal electrode montages and current densities for each electrode can be identified to ensure high focality at the grey matter level (Khorrampanah et al., 2020). Efficient identification of optimal tES parameters based on individual baseline abilities can enhance intervention efficacy (van Bueren et al., 2021) and define these parameters in real‐time based on subjective performance (Lorenz et al., 2019). Also, results evaluation can be implemented by these approaches: resting‐state magnetic resonance imaging data have shown that tDCS can effectively engage specific anatomical tracts, with classification algorithms used to identify this engagement (Shinde et al., 2023).

In a near future, the patient journey will begin with a comprehensive psycho‐neural assessment. Utilising state‐of‐the‐art imaging, the team will map intricate neural circuits and identify specific markers associated with cognitive disorders. Genetic profiles add an extra layer of precision, revealing individual variations that influence treatment responses. Integrated real‐time neural monitoring and adaptive algorithms enable the precise modulation of electrical currents based on individual neuro‐cognitive states. Such individual ‘fingerprints’ approach will aim to optimise the efficacy of tES by tailoring stimulation parameters to each person's specific characteristics. We can say that clinical interventions are evolving into dynamic, data‐driven approaches.

### Variability and recovery capacity of brain functions

Discussing how to apply tES, we have been focused on the use of complex technological aspects, which should not distract us from the important resources and high adaptive capacity of the nervous system. Especially in the cognitive neuropsychological field, we must consider the patient variability with special attention. It has been suggested that variability provides insights into predicting recovery capacity after brain damage (Seghier & Price, 2018) but also facilitates the activity of alternative networks that can sustain similar tasks. We should not see variability as a nuisance that reduces expected results, but as a resource for patient and function rehabilitation. Recovery of a function following a lesion/pathology might be due to many factors. It could be a gradual readjustment of an intact but functionally suppressed area, and this would be the simplest instance. Applying tES to a function‐dedicated network can promote readjustment through a potentiation‐like phenomenon, potentially aiding in function recovery. However, in some patients, the neurostimulation and recruitment or rebalance of different networks may be used to accomplish the impaired function and compensate for the deficit. This means that brain areas, due to their ability to adapt, can take over functions they did not typically perform before, although they may have contributed to these functions. Learning is a property of neuronal networks, where plasticity has a variability‐based signature, so variability can favour the activation of a different circuit to perform a function. Without such variability, there would be no room for learning, because all neural networks would respond in a fixed way to a precise activation and would be poorly activated by ‘stimuli/tasks’ that vary from the basic activation (Ilan, 2023). Therefore, we must consider the variability of patients' responses as a resource, and we must exploit it to lay the foundations for more effective treatments.

## CONCLUSIONS

As clinicians, we are interested in exploiting the efficacy of tES in various aspects of cognitive rehabilitation, such as memory enhancement, language improvement and other skill rehabilitation. Many research studies and clinical trials are ongoing to investigate the specific protocols, optimal parameters and target areas for tES application to maximise therapeutic benefits. Nevertheless, to reach this goal, it is important that a shift in approach takes place.

We are witnessing an increased personalisation of prevention, diagnosis and intervention throughout the clinical care trajectory. This involves discovering predictive, diagnostic and prognostic biomarkers to characterise patients for targeted interventions. Capturing patient ‘fingerprint variability’ relevant population patterns and precisely manipulating task‐induced neural activity constitute a viable approach. It could catalyse next‐generation artificial intelligence‐guided neurointerventions, potentially enhancing the potential of rehabilitation of higher‐order functions mediated by distributed neural networks (Gupta et al., 2023).

Nevertheless, this also implies a network collaborative approach between neuropsychologists, neurologists, cognitive/speech therapists, cognitive neuroscientists, engineers and other field‐specific professionals as elements of vital importance to redefine clinical interventions through a refined approach that harnessed the latest breakthroughs in understanding and treating the brain. Moreover, the full sharing of the outcome data can help us in training neural network systems and better understand which parameters modulate the response or recovery by the well‐characterised patient. Sharing FAIR data and running collaborative projects to build consensus‐based evaluation and intervention protocols will be a significant step forward to get off the ground and offer the patient the most likely approaches to maximise benefit and reduce harm (Kaplan & Irvin, 2015).

## AUTHOR CONTRIBUTIONS

**Carlo Miniussi:** Writing – review and editing. **Maria Concetta Pellicciari:** Writing – review and editing.

## CONFLICT OF INTEREST STATEMENT

None of the authors have a conflict of interest to disclose.

## Data Availability

Data sharing not applicable to this article as no datasets were generated or analysed during the current study.
